# Gradient-Hierarchic-Aligned Porosity SiOC Ceramics

**DOI:** 10.1038/srep41049

**Published:** 2017-01-20

**Authors:** Cekdar Vakifahmetoglu, Damla Zeydanli, Murilo Daniel de Mello Innocentini, Fernanda dos Santos Ribeiro, Paulo Renato Orlandi Lasso, Gian Domenico Soraru

**Affiliations:** 1 Department of Mechanical Engineering, Istanbul Kemerburgaz University, 34217, Istanbul, Turkey; 2 Department of Chemistry, Istanbul Technical University, 34469, Istanbul, Turkey; 3 Course of Chemical Engineering, University of Ribeirão Preto (UNAERP), 14096-900 Ribeirão Preto, SP, Brazil; 4 Embrapa Agricultural Instrumentation, P.O. Box 741, 13560-970 São Carlos, SP, Brazil; 5 Dipartimento di Ingegneria Industriale, Università di Trento, Via Sommarive 9, 38123 Trento, Italy

## Abstract

This work describes a simple technique to produce porous ceramics with aligned porosity having very high permeability and specific surface area. SiOC-based compositions were processed from blends of three types of preceramic polymer and a catalyst, followed by curing and pyrolysis. The heating applied from the bottom of molds promoted the nucleation, expansion and rising of gas bubbles, and the creation of a ceramic matrix with axially oriented channels interconnected by small round pores. The samples were analyzed by SEM, tomography, BET, water immersion porosimetry and permeation to gas flow. The resulting bodies presented levels of open porosity (69.9–83.4%), average channel diameter (0.59–1.25 mm) and permeability (0.56–3.83 × 10^−9^ m^2^) comparable to those of ceramic foams and honeycomb monoliths, but with specific surface area (4.8–121.9 m^2^/g) typical adsorbents, enabling these lotus-type ceramics to be advantageously used as catalytic supports and adsorption components in several environmental control applications.

Porous materials, especially ceramics, have been successfully used in many technological processes such as insulation (buildings, machines, etc.), filtration and purification (molten metals, gases, wastewater, etc.) and catalysis^1^,^2^. Various processing routes (etching, sacrificial templating, direct foaming, replica, additive manufacturing, etc.) have been explored for the production of porous ceramics, and several reviews are available on the subject^1^,^3^,^4^,^5^,^6^. In a recent review, application based properties of those materials with respect to microstructural features have also been published^7^. It was demonstrated that the aligned porosity (sometimes called unidirectional, lamellar or lotus type porosity) could have an important and beneficial role in some applications where better thermal insulation (when the heated surface is orthogonal to aligned porosity) or enhanced fluid/gas flow (when the flow direction is parallel to aligned porosity) is desired^2^,^7^,^8^.

In the recent years, production of permeable, aligned porosity ceramics has attracted increasing attention, and several methods have been proposed. Okada *et al*.^9^ summarized these methods as anodic oxidation, templating (with synthetic, or natural components such as wood), unidirectional solidification, extrusion, and other methods (bubbling, application of magnetic fields, and filament winding). It is worth nothing that among all these studies, unidirectional solidification is the most commonly employed method, which can be categorized as *unidirectional solidification of melts* and *freeze casting*. While the first one has mostly been applied to metallic materials^2^, the latter was extensively investigated to form ceramic materials from suspensions at low temperatures^10^.

In freeze casting, a freezing medium (water, camphene, etc.) serves as a template to be sublimed, resulting in a dendritic, aligned channeled matrix^7^,^10^. Although rare, preceramic polymers have also been investigated in the production of aligned porosity ceramics by freeze casting^6^,^11^,^12^. In the unidirectional solidification process, generally a molten metal is pressurized in a gas atmosphere (e.g. hydrogen, nitrogen, etc.) and during solidification the evolving gas (gases have lower solubility in the solid than molten phase) creates such directional pores^2^,^9^. While unidirectional porosity metals also called *Gasar*^13^ or *lotus-type*^2^ (due to the similarity of the final pore morphology with lotus root) have been fabricated following this approach, only a few ceramic composition (SiC, ZrO_2_/MgO, MgAl_2_O_4_/Al_2_O_3_, Al_2_O_3_, mullite)^9^,^14^,^15^,^16^,^17^,^18^,^19^ were manufactured yet. Recently following the same method, preceramic polymer blends were crosslinked in Teflon lined stainless steel autoclaves and pyrolyzed, resulting in samples with a unidirectional porosity reaching around 65%^20^. Although it is a simple process, such solidification technique inherently limits the size of the sample that can be produced to a gas chamber size (e.g. autoclave), and as in the case of freeze casting, raises difficulties in controlling the pore size/shape of the produced sample^9^.

In order for a porous component to possess a wide range of desirable characteristics, such as rapid transport of fluids and gases, low pressure drop, high selectivity, fast uptake and release, etc., it should comprise a high specific surface area (SSA) together with interconnected cellular (micron range) framework, i.e. hierarchical porosity^4^. In these aforementioned methods, the goal was merely to form aligned porosity materials, but the formation of components that facilitate tunable aligned porosity together with high surface area was never explored. In this paper, we present a facile technique, not requiring any freezing medium or specialized equipment (e.g. autoclave) apart from a simple hot-plate, to form hierarchically porous monolithic SiOC ceramics, having interconnected aligned porosity. Characterizations included evaluation of bulk and superficial properties and also permeability to air flow.

## Results

### Morphological characterization

**Figure 1:**
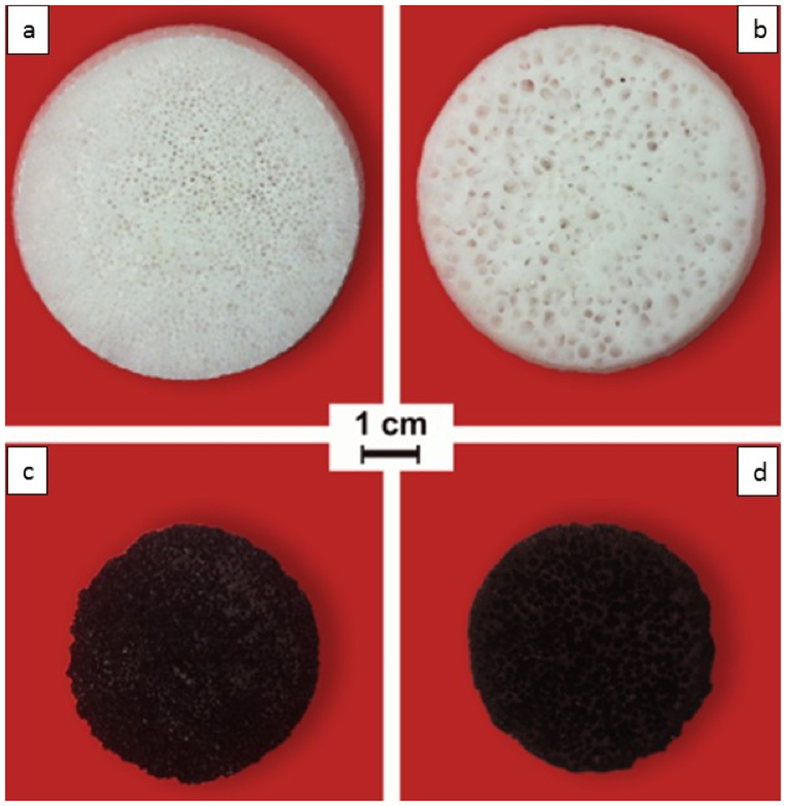
Digital photographs demonstrate the gradient porosity changing from bottom to top side for both thermoset and pyrolyzed sample. In the top, photos show white colored thermoset (cured) samples while in the photos given below pyrolyzed, so called “black glass”, samples can be seen. Photos were taken; (**a**) from the bottom side of the cured thermoset; (**b**) from the upper side of the cured thermoset; (**c**) from the bottom side of the pyrolyzed; and (**d**) from the upper side of the pyrolyzed sample.

Photographs of sample L1, both in cured and pyrolyzed state are given in Fig. 1. It was observed that the pores at the bottom of the samples (Fig. 1(a and c)) were smaller compared to those at the top (Fig. 1(b and d)) causing an aligned but also gradient porosity. Polymer derived ceramics with porosity gradient (but not aligned) have already been observed by other researchers^21^,^22^,^23^. Although the produced samples were 4 cm diameter and 1.5 cm thickness after pyrolysis, with the described procedure, it is possible to manufacture complex shapes (the samples can easily be machined in cured state) and much larger diameters (e.g. cylindrical samples in cured state with 10 cm diameter with no flaws or cracks were formed but due the furnace’s tube diameter, these samples could not be pyrolyzed in such dimension). Note that the thickness is one of the main variable in this process, as it affects the temperature gradient applied across the sample and thus the gas evolution and the ultimate morphology of created channels; however, this issue needs to be assessed in further studies.

**Figure 2:**
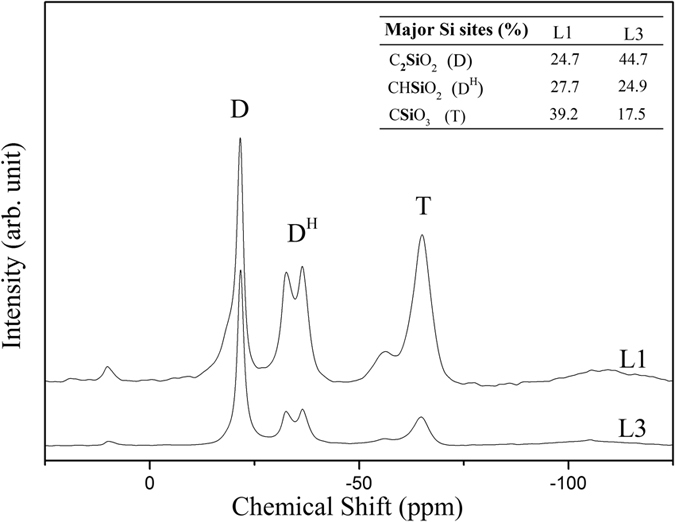
^29^Si MAS-NMR spectra recorded on L1 and L3 precursors. Inset refers to the percentage of Si sites obtained by decovoluting the ^29^Si MAS NMR spectra. Difference to 100% is due to the presence of small amounts of C_3_SiO (M units at ca 10 ppm) and SiO_4_ (Q units at ca −108 ppm).

As previously shown, the PHMS polymer can be crosslinked via hydrosilation when there are additional vinyl-containing precursors or via hydrosilylation/dehydrocoupling when there is water in the system^24^,^25^,^26^,^27^. The latter case results in the formation of hydrogen (H_2_) gas that can be used as to “self blow” the system to obtain porous components^23^,^24^,^28^,^29^. In the present experiments, the heat applied from the bottom of the aluminum mold initiated the crosslinking reactions, simultaneously the gas bubbles nucleated probably with the aid of LDH particles (both as a surface and water including source), expelled from the heated surface and moved into the cooler zone till reaching to the upper surface (atmospheric). Additional experiments (not shown for the brevity) in which the crosslinking was conducted in the oven (uniform heating) instead of hot-plate directional heating, foams with no aligned porosity were obtained^22^,^24^,^28^. This implies that the formation of aligned channels was caused by the temperature gradient from bottom to top resulting in simultaneous curing and gas release together with the coalescence of gas bubbles during raise, yielding larger channels on the top. Investigation of the molecular structure of the crosslinked precursor has been performed recording ^29^Si MAS NMR spectra (Fig. 2). This technique reveals the local environments around the Si atoms and helps understanding the crosslinking mechanisms active for the studied system.

The spectra reported in Fig. 2 for the L1 and L3 samples show the presence of three main Si sites: at 22 ppm C_2_**Si**O_2_ in which Si is bonded to two carbon and two oxygen atoms, at 32–36 ppm CH**Si**O_2_ where Si is bonded to one carbon, one hydrogen and two oxygen atoms; at 64 ppm in which Si is bonded to one carbon and thee oxygen atoms^30^. According to the usual notation in silicon chemistry the sites can be referred as D, D^H^ and T respecively. D^H^ sites are the Si sites present in the starting PHMS precursors and their presence in the crosslinked precursor indicate the incomplete consumption of the starting Si-H bonds. D sites are present in the starting PDMS as well as in the TMTVS but can also be the results of the hydrosylilation reaction between the Si-H groups of PHMS and the Si-CH = CH_2_ moieties of the PDMS/TMTVS. Finally T sites unambiguously demonstrate the occurrence of the dehydrocoupling reactions during polymer crosslinking which leads to the transformation of a Si-H bond into a Si-O bond. A quantitative analysis of the various Si sites, performed by deconvoluting the individual components, gave the results reported in the inset of Fig. 2. Sample L3 which has been synthesized using a higher amount of PDMS compared to L1 consistently shows more D units.

**Figure 3:**
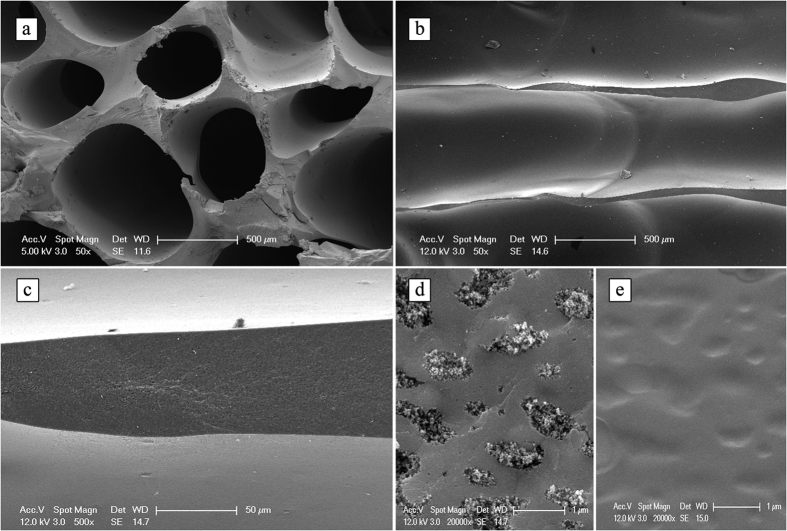
SEM images taken from the fracture surfaces of pyrolyzed sample: (**a**) pores from top portion; (**b**) cross section showing the aligned channels; (**c**) channel strut detail; (**d**) magnified strut view; (**e**) closed magnification view of pore surface.

SEM images of the samples are given in Fig. 3(a–e). The channels formed were generally larger than 500 μm when measured from the top section of sample, instead at the bottom part they were getting narrower, see later for detailed pore analysis. Aligned channels (Fig. 3(b and c)) were seperated with 50–100 μm ranged struts, while the strut (i.e., fractured matrix) presented pores with around 1 μm diameter (Fig. 3(d)) and dense channel surfaces (Fig. 3(e)).

**Figure 4:**
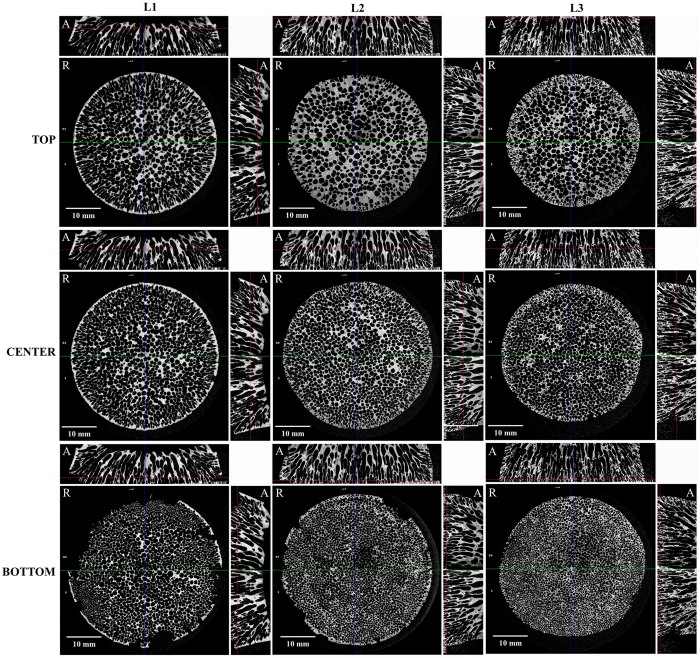
Selected tomografic images of radial (R) and axial (A) slices at the top, center and bottom of SiOC samples L1, L2 and L3.

Tomographic 2D images of disk samples L1, L2 and L3 are given in Fig. 4. It is observed in the radial slices (R) that channels present circular cross-section in the center but are elongated near the borders, due probably to the temperature gradient from center to edges. On the other hand, the axial slices (A) show aligned channels connecting both faces of the disks. The number of channels visually decreased from bottom to top while their cross-sectional area increased in the same direction, which confirms the coalescence of gas bubbles and merging of channels.

**Figure 5:**
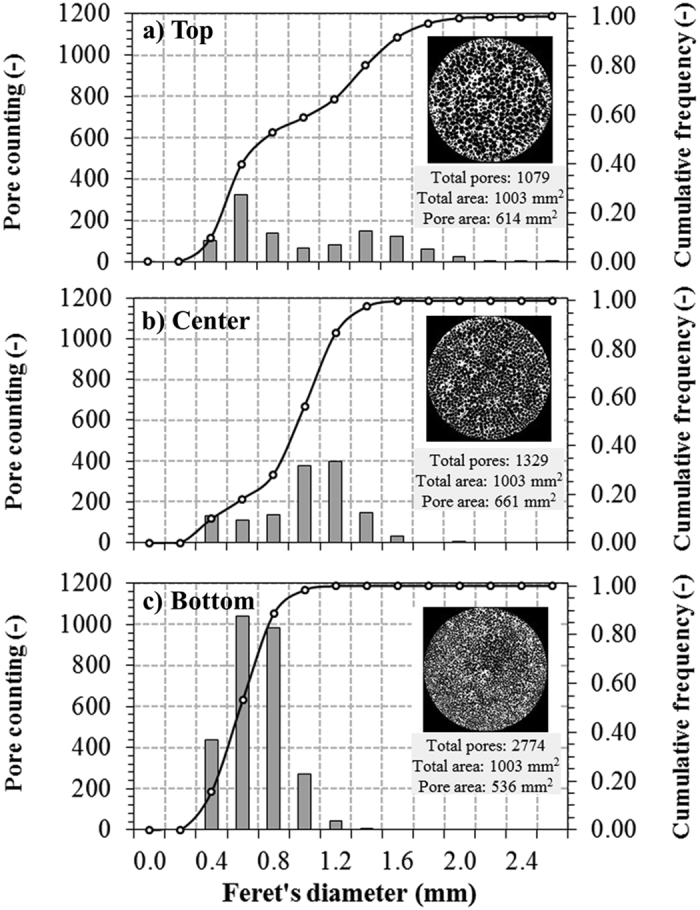
Channel diameter distributions of selected radial tomographic slices of SiOC sample L3: (**a**) top; (**b**) center; (**c**) bottom.

**Figure 6:**
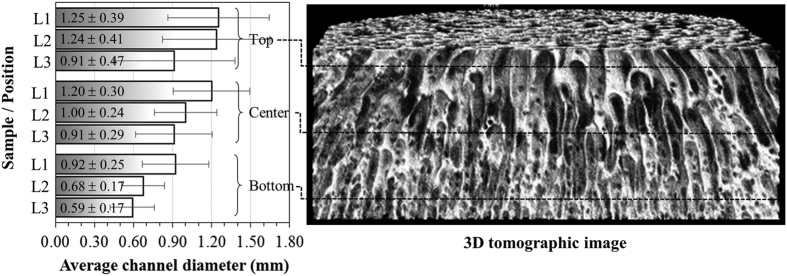
Average channel diameters of selected radial tomographic slices at the top, center and bottom of SiOC samples L1–L3.

Typical pore counting on selected radial tomographic slices are illustrated in Fig. 5 for sample L3. The Feret’s diameter, defined as the averaged distance between pairs of parallel tangents to the projected outline of the object, was chosen to specify the cross-sectional size of channels in the 2D images. For a same analyzed area (1003 mm^2^), the counting decreased more than 60% (from 2774 in the bottom to 1079 in the top) due to merging of channels. Accordingly, the channel diameter distribution was shifted to higher values. Observed diameters were in the range 0.28–2.9 mm. It is also worth noting that the cross-sectional void area increased in the upward direction, which indicates that the rising bubbles not only merged but also expanded before solidification of the matrix. The same trends were observed for samples L1 and L2 and the average channel diameters are given in Fig. 6. It is possible to observe in the reconstructed 3D image many small round pores that connect adjacent vertical channels along their entire length. These pores are probably resulting from small bubbles that were entrapped during the solidification of the ceramic suspension, and represent a unique feature, as they create an effective path for permeation and diffusion of fluids also in the radial direction. Compared to other aligned porosity ceramics, the average diameter of channels of SiOC samples (0.59–1.25 mm) are clearly higher than of those processed by ice-templating (2.9–19 μm)^31^,^32^ and by inclusion of polymeric fibers as sacrificial fillers (9–43 μm)^8^,^32^. On the other hand, channel size distributions in samples L1–L3 were much more homogeneous than those observed in biomorphic SiC prepared from hardwood precursors, where the vascular transportation system in the tissue creates naturally a bimodal diameter distribution of large vessels surrounded by very small channels^32^,^33^,^34^,^35^.

### Porosity and surface area characterization

**Figure 7:**
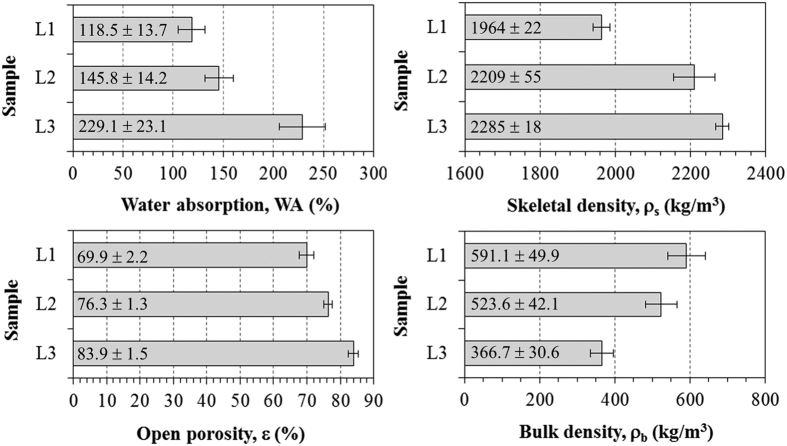
Bulk properties of SiOC porous samples obtained by water immersion tests.

Results of water immersion porosimetry are given in Fig. 7. SiOC foams presented skeletal density ρ_s_ (1964–2285 kg/m^3^) smaller than alumina (3900–4100 kg/m^3^), silicon carbide (3100–3200 kg/m^3^), and mullite (3100–3300 kg/m^3^) and traditional refractory (bricks) made from clay minerals (2500–2900 kg/m^3^), the most common components of refractory porous ceramics, but were similar to activated carbons (1900–2400 kg/m^3^) used in adsorption applications^32^,^36^,^37^. The observed increase in ρ_s_ for sample L3 can be explained by the elimination of closed pores in the matrix with the help of PDMS molecular spacer^25^, which leads, during pyrolysis, to the formation of connecting meso-sized-channel in between what before was closed porosity.

The open porosity ε (69.9–83.4%) was within the range found for reticulated foams (60–95%)^32^,^33^ and much higher than that of packed beds of loose monosized particles (35–45%)^32^,^38^. Combination of low skeletal density and high porosity resulted in SiOC monoliths of low bulk density ρ_b_ (591.1–366.7 kg/m^3^), typical of lightweight porous ceramics^16^,^39^. The increases in ε and ρ_s_ and the decrease in ρ_b_ were due to the increase of PMDS, which acted as a templating agent since this polymer decomposes completely and leaves behind a porosity whose size depends on the molecular weight^25^.

**Figure 8:**
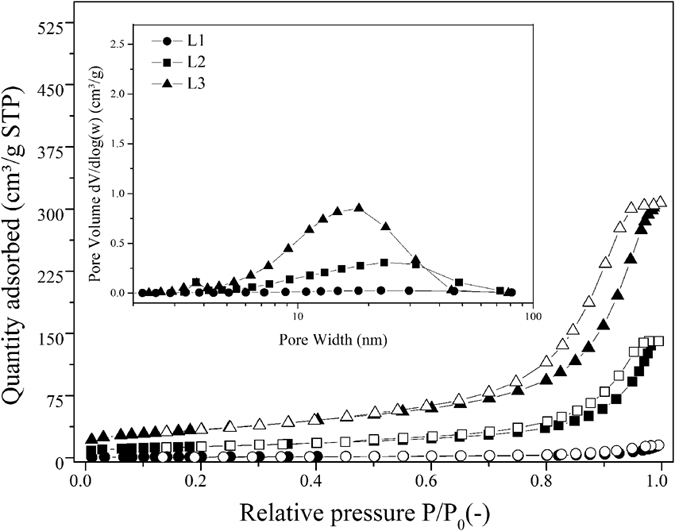
N_2_ sorption isotherms of SiOC porous samples. The inset refers to the PSDs calculated from the desorption branches of the isotherms. While filled symbols show adsorption branches of isotherms, empty ones for the same symbol show desorption branches.

Complementary information on the microstructure of the porous SiOC samples were obtained from the N_2_ sorption analysis reported in Fig. 8 and Table 1. The pore size distribution curves reported in the inset of Fig. 8 show the presence of mesopores in the size range 10–50 nm. It is known that during pyrolysis of preceramic polymers blends which includes similar PDMS, decomposition of such precursor forms mesopores pores in the size range 10–100 nm^25^ which is consistent with our observations. Instead the porosity arising from the blowing agent (H_2_ formed via dehydrocoupling) is much larger, in the hundred microns–/millimeter range.

Accordingly, with the increase in PDMS amount, the SSA values increased, which is consistent with the observed increase in apparent porosity, as previously shown in Figs 6 and 7. When 1PHMS:0.25PDMS mixture was used, a SSA of 4.3 m^2^/g was obtained, but doubling PDMS in the blends resulted in 48.6 m^2^/g and eventually 121.9 m^2^/g with 1PHMS:1PDMS. These SSA values are within the range observed for adsorbent materials^32^,^40^. From the isotherms shown in Fig. 8 it can be seen that while L1 did not present any noticible meso-macroporosity with the increase in PDMS amount, for L2 and L3, a clear hystheresis loop appeared in the adsorption-desorption isotherms above P/P_0_ = 0.8, indicating the presence of large-mesopores/macropores. Similar surface area enhancement due to PDMS decomposition and so additionally formed mesoporosity was also observed in other studies^25^,^41^.

In Fig. 8 (inset), the pore size distribution (PSD) obtained by the N_2_ sorption tests are given. Sample with lowest PDMS amount (L1) shows almost no micro-meso-porosity, but increasing the PDMS resulted in the formation of a broad peak located around 20 nm, analogous to previous observations^25^. The intensity of such peak increased when the PDMS amount increased compared to PHMS, as can be seen. In Table 1, the mean pore diameters are calculated by d = 4 V/A, where V is the total pore volume and A is the BET surface area^42^,^43^. This model for the average pore size assumes that the pores are cylindrical and open at both ends; therefore this mathematical expression does not include much physical meaning unless the data come from mono-modal and narrowly-distributed pores, which is not the case for present study.

### Permeability evaluation

**Figure 9:**
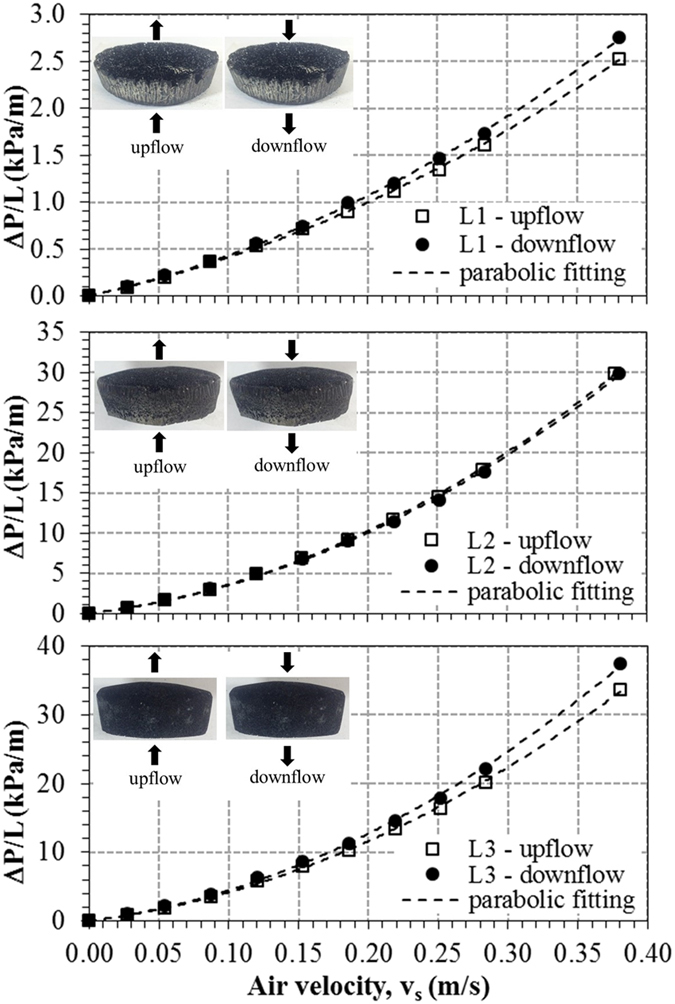
Normalized pressure drop curves in the airflow experiments for SiOC samples L1, L2 and L3.

Figure 9 displays the length-normalized pressure drop curves (ΔP/L) for samples L1 to L3 obtained from airflow tests at room temperature and face velocities (v_s_) up to 0.4 m/s. Tests were performed in two orientations (up-flow and down-flow) along the axial direction of the disks. Forchheimer’s equation (Eq. 1) was suitably fitted to experimental data (R^2^ > 0.99 in all cases), as can be seen by the dashed lines, which confirms the validity of the parabolic model to describe the relationship between pressure drop and air velocity. In this situation, simplification to Darcy’s law is not recommended, as the contribution of the inertial term [ρv_s_^2^/k_2_] of Eq. (1) on total pressure drop exceeded 50% for v_s_ = 0.40 m/s. Comparatively, the pressure drop level increases from L1 to L3, which was consistently confirmed in the three samples tested for each composition. Despite the gradient of porosity and pore diameter in the axial direction, no important effect was observed in pressure drop by inverting the flow orientation.

**Figure 10:**
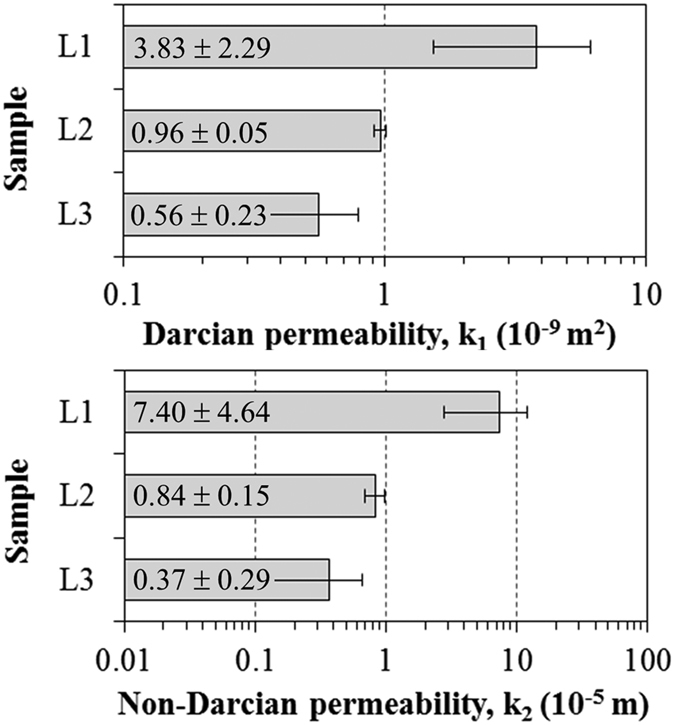
Permeability coefficients of SiOC foams retrieved from airflow experiments.

The permeability coefficients of SiOC foams were retrieved from fitting of Forchheimer’s equation to the pressure drop curves in Fig. 9. The resulting values of k_1_ and k_2_ are given in Fig. 10. The great advantage of using these parameters to represent the permeation behavior of a porous medium is that they are only dependent on the pore descriptors (size, volume, morphology, interconnectivity, etc.) and therefore may be used to simulate the ∆P × v_s_ profile for other fluids or flow conditions, provided that ρ and μ in Eq. (1) are corrected in accordance with the temperature and pressure. Any change in the processing variables that results in a decrease in the interconnected porosity and pore size, or in an increase in tortuosity and roughness of the channel walls will lead to lower values of k_1_ and k_2_, even though with different intensity ranges. In the present case, despite the increase in open porosity (69.9 to 83.4%) observed in Fig. 7, the decrease in the channel diameter (1.25 to 0.59 mm), as shown in Fig. 6, was the decisive factor for the reduction of 85% in k_1_ and 95% in k_2_ from L1 to L3. The benefits of a higher specific surface area and lower bulk density promoted by the increase in the PDMS amount were thus partially hindered by the higher difficulty to permeate fluids. Nevertheless, the k_1_ level of SiOC foams (0.56–3.83 × 10^−9^ m^2^) is at least two orders of magnitude higher than that reported for biomorphic wood-based ceramics and other porous ceramics prepared with sacrificial fillers^32^,^44^,^45^.

**Figure 11:**
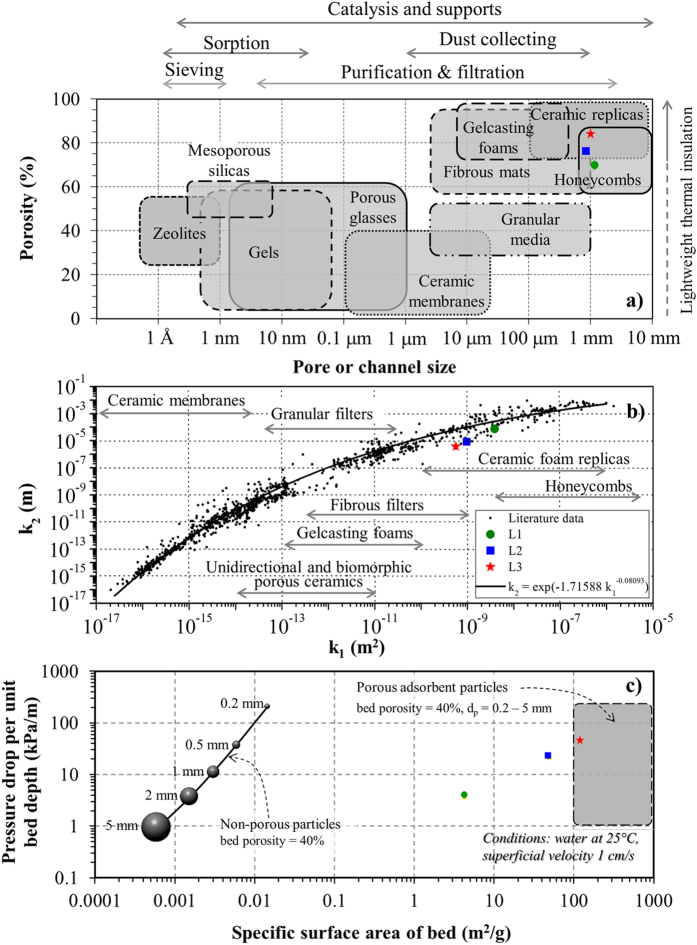
Maps of properties and applications of porous ceramics with location of SiOC samples L1, L2 and L3 (note that data represented are average values, and such values for SiOC ceramics obtained from 2 different samples in 2 flow orientations): (**a**) porosity map adapted from Okada *et al*.^9^,^32^; (**b**) permeability map adapted from Innocentini *et al*.^33^,^34^,^35^,^47^; (**c**) specific surface area map, adapted from Boger *et al*.^48^.

The aforementioned features of SiOC lotus-type foams enable these structures to be used in a variety of applications^7^,^16^,^39^,^46^. In Fig. 11, a set of three maps of properties-applications of porous materials were gathered from the literature, showing the location of compositions L1, L2 and L3. The map in Fig. 11(a), adapted from Okada *et al*.^9^,^32^, identifies several groups of porous materials according to their common porosity and pore size levels and the usual application. Samples are included in the range of honeycombs and ceramic replicas, used for molten metal filtration and as supports for catalytic soot filtration in diesel engines^7^,^39^,^46^. The map in Fig. 11(b), adapted from Innocentini *et al*.^33^,^34^,^35^,^47^, confirms the high permeability level of SiOC foams, comparable to honeycombs and ceramic replicas and much higher than other biomorphic unidirectional porous ceramics. The map in Fig. 11(c), adapted from Boger *et al*.^48^ reveals that lotus-type SiOC foams intrinsically present much higher specific surface area and lower pressure drop than packed beds of non-porous loose particles, and are nearly equivalent of typical adsorbents used in removal of contaminants in water and gas streams^49^,^50^,^51^,^52^. It is worth noting that honeycombs and ceramic replicas are also used in such adsorption/catalytic applications because of their low pressure drop level, but they need to be pre-treated with coatings to increase the active surface area. SiOC lotus-type foams produced by the one-pot synthesis method described here directly have such features, which can be further optimized by manipulation of processing variables, for instance to functionalize the surface area for selective adsorption or catalytic action^33^,^34^,^35^,^40^,^47^,^48^.

## Discussion

An inexpensive and simple technique based on the blends of preceramic polymers and unidirectional heating followed by curing and pyrolysis was applied to generate SiOC bodies with aligned porosity and high specific surface area. The process is robust since it works with different Si-H/C = C molar ratios, which implies different amounts of generated H_2_. However, the structural and physical analyses revealed that, in order to get the desired microstructure, and in particular to control the volume and size of axially oriented channels (having average diameter in between 0.59–1.25 mm), the proportion of polydimethylsiloxane (PDMS) in the blend was the key processing parameter. As a result, a porous structure (open porosity reaching to 83.4%) with surface area (121.9 m^2^/g) comparable to commercial adsorbents but with pore size and permeability levels comparable to honeycombs and ceramic foam replicas was created. Such versatility in processing and in properties can be useful to produce porous components for demanding environmental applications.

## Methods

### Materials

Three types of polysiloxanes were used: a linear polymethylhydrosiloxane (*Gelest, PHMS, MW*~*2100*–*2400, 30*–*45 cST, CAS: 63148-57-2, Gelest, Morrisville, PA, USA*) having Si-H bonds, a vinyl-terminated polydimethylsiloxane (*PDMS, MW:62700, 10000 cSt, CAS: 68083-19-2, Gelest, Morrisville, PA, USA*) and a cyclic 2,4,6,8-tetramethyl-2,4,6,8- tetravinlycyclotetrasiloxane (*TMTVS 97*%, *Alfa Aesar, Ward Hill, MA, USA, CAS: 2554-06-5*) with Si-C = C moieties. Platinum - divinyltetramethyldisiloxane complex, ~Pt 2% in xylene (*CAS: 68478-92-2, Sigma-Aldrich, St. Louis, MO, USA*) was used as catalyst for the curing reactions. Hydrotalcite, (*LDH with a formula (Mg*_*6*_*Al*_*2*_(*CO*_*3*_)(*OH*)_*16*_*·4H*_*2*_*O*), *CAS Number 11097-59-9, Sigma-Aldrich, St. Louis, MO, USA*), was used as received.

### Manufacture of aligned porosity SiOC foams

Firstly, PHMS/LDH/PDMS/cyclic TMTVS were put into a glass beaker in the weight ratio of 1/0.055/0.25/0.055 (as sample L1), 1/0.055/0.5/0.055 (L2), and 1/0.055/1/0.055 (L3), i.e. keeping all others in the same ratio but increasing the PDMS amount. The blend was mixed under magnetic stirring at room temperature (RT) for 5 min at 500 RPM until the homogeneous mixture was achieved. Upon homogenization, 100 ppm by weight of Pt relative to PHMS was added into the mixture in a dropwise manner. The blend was further mixed at 500 rpm for 25 min and transferred into aluminum molds standing on the hot plate operating at 200 °C for curing overnight.

All the samples were pyrolyzed in a tubular furnace (*PROTERM PTF 16*/*75*/*450, Ankara, Turkey*) at 1300 °C with a heating rate of 2 °C/min and 1 h dwell time at the maximum temperature under N_2_ flow (100 mL/min). The furnace was purged for 5 h with flowing N_2_ before pyrolysis. At least three samples of each composition L1, L2 and L3 were produced for characterization.

### Characterization

The molecular structure of the crosslinked preceramic samples was investigated recording ^29^Si MAS NMR spectra with a Bruker 300 WB instrument (*Bruker Instruments, Karlsruhe, Germany*) operating at a proton frequency of 300.13 MHz. NMR spectra were acquired with single pulse (SP) sequence under the following conditions^29^: Si frequency: 59.60 MHz, π/4 pulse length: 2.25 μs, recycle delay: 150 s, 4 k scans. Samples were packed in 4 mm zirconia rotors, which were spun at 5 kHz under air flow. Q8M8 was used as external secondary reference.

The morphological features of samples were analyzed from fresh fracture surfaces using a scanning electron microscope (*FEI-Philips XL30 ESEM-FEG*, the Netherlands) after 10 nm Pt film deposition by sputtering. Powders were analyzed by Nitrogen (N_2_) gas adsorption. The isotherms were collected at 77 K using an ASAP 2010 (*Micromeritics, Norcross, GA, USA*) after sample degassing at 200 °C for minimum 4 h before analysis.

Specific surface area (SSA) was calculated from a BET (Brunauer, Emmet and Teller) analysis in the *P*/*P*_0_ range of 0.05–0.30 with minimum of five data points. The pore size distributions in the mesopores range were obtained from the desorption branch of the isotherm through the BJH (Barret, Joyner and Halenda) analysis.

The water displacement method, based on the Archimedean principle (ASTM C20-00)^53^, was used to evaluate the water absorption (WA), open porosity (ε), bulk density (ρ_b_) and skeletal density (ρ_s_) of samples.

A high-resolution X-ray micro-CT system (*SkyScan, model 1172, Aartselaar, Belgium*) was used to analyze the two- and three-dimensional morphological parameters of whole disk samples (≈40 mm diameter and ≈10 mm thickness). For each sample, about 920 radial slice images (pixel size of 11.98 μm) were reconstructed to 8-bit BMP files (3968 × 3968 pixels) using the SkyScan CT analyzer software package. Pore counting and pore size distribution (Feret’s diameter) were obtained with the software ImageJ 1.50i from binarized slice images (circular area of ≈1000 mm^2^) selected at the top, center and bottom of each sample.

Experimental evaluation of permeability was carried out in a laboratory-made apparatus, with tests performed in steady-state regime with dry airflow at room conditions (T ≈ 29 °C, P_atm_ ≈ 94.7 kPa) on 2 specimens of each composition. The disk sample was laterally sealed within a cylindrical chamber that provided a circular flow area (A_flow_) of 3.37 cm^2^, for a useful medium diameter of 2.07 cm. The pressure drop across the specimen (P_i_ − P_o_) was measured by a digital micro-manometer (*Dwyer Mark III, model 475, Michigan, USA*) in response to variations in the air volumetric flow rate Q, controlled by a needle valve and measured with a rotameter (*Conaut, São Paulo, Brazil*) open to the atmosphere. Flow rate (Q) was corrected to the value at sample exit (Q_o_) and finally converted to superficial velocity by v_s_ = Q_o_/A_flow_. In order to assess flow anisotropy, tests were performed with air stream sequentially in opposite axial orientations: (a) up-flow and (b) down-flow by changing valves and connections. Further details of the setup are described elsewhere^32^,^33^,^34^,^35^.

Permeability parameters were retrieved from experimental data and fitting of Forchheimer’s equation, an empirical relationship well accepted in the literature to express the parabolic dependence of pressure drop (∆P) with the resulting superficial or face velocity (v_s_) of fluid through the medium^33^,^34^,^35^.


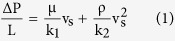


for which L is the medium length or thickness along the macroscopic flow direction, and μ and ρ are respectively the viscosity and density of air. The parameters k_1_ and k_2_ are respectively known as Darcian and non-Darcian permeability coefficients, in reference to Darcy’s law, which establishes a linear dependence between ∆P and v_s_. These coefficients are only dependent of the porous structure and weigh the contributions of viscous and inertial losses on the total pressure drop in Eq. (1). Notably, k_1_ is expressed in square length dimensions, while k_2_ is expressed in length dimensions to maintain dimensional consistency in Eq. (1).

For compressible flow of gases, ∆P in Eq. (1) must be calculated by Eq. (2) given by:


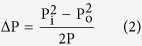


in which P_i_ and P_o_ are, respectively, the absolute fluid pressures at the entrance and exit of the medium. P is the pressure for which v_s_, μ and ρ are measured or calculated (in this work P = P_o_).

The collected data set (P_i_, P_o_ and v_s_) for each test was fit and the permeability parameters were then calculated from the fitted constants a (k_1_ = μ/a) and b (k_2_ =  ρ/b) of the Forchheimer’s equation (Eq. (1)).

## Additional Information

**How to cite this article**: Vakifahmetoglu, C. *et al*. Gradient-Hierarchic-Aligned Porosity SiOC Ceramics. *Sci. Rep.* **7**, 41049; doi: 10.1038/srep41049 (2017).

**Publisher's note:** Springer Nature remains neutral with regard to jurisdictional claims in published maps and institutional affiliations.

## Figures and Tables

**Table 1:** Summary of the N_2_ sorption test results from all samples.

Sample Code	Surface area - BET (m^2^/g)	Micropore volume (cm^3^/g)	Pore volume (cm^3^/g)	Pore size (nm)
			BJH Desorption cumulative volume of pores	BJH Desorption average pore width (4 V/A)
L1	4.3	0.001	0.023	21.4
L2	48.6	0.001	0.218	17.9
L3	121.9	0.004	0.473	15.5
